# In vitro Fermentation of Polysaccharides from *Aloe vera* and the Evaluation of Antioxidant Activity and Production of Short Chain Fatty Acids

**DOI:** 10.3390/molecules24193605

**Published:** 2019-10-07

**Authors:** Antonio Tornero-Martínez, Rubén Cruz-Ortiz, María Eugenia Jaramillo-Flores, Perla Osorio-Díaz, Sandra Victoria Ávila-Reyes, Guadalupe Monserrat Alvarado-Jasso, Rosalva Mora-Escobedo

**Affiliations:** 1 Instituto Politécnico Nacional, ENCB, Campus Zacatenco. Miguel Othón de Mendizábal 699, Alcaldía G.A. Madero, Ciudad de México C.P. 07360, CDMX, Mexico; torneroayora@gmail.com (A.T.-M.); rub66.63@gmail.com (R.C.-O.); Jaramillo_flores@hotmail.com (M.E.J.-F.); 2 Instituto Politécnico Nacional, CEPROBI, Carretera Yautepec-Jojutla, Km. 6, Yautepec C.P. 62731, Morelos, Mexico; posoriod@gmail.com (P.O.-D.); Sandra_victory@yahoo.com (S.V.Á.-R.); gpemonserratjasso@gmail.com (G.M.A.-J.); 3 CONACYT—Instituto Politécnico Nacional, CEPROBI, Carretera Yautepec-Jojutla, Km. 6, Yautepec C.P. 62731, Morelos, Mexico

**Keywords:** *Aloe vera*, *Aloe vera* polysaccharides, in vitro fermentation, SCFAs, antioxidant capacity

## Abstract

Soluble or fermentable fibre has prebiotic effects that can be used in the food industry to modify the composition of microbiota species to benefit human health. Prebiotics mostly target Bifidobacterium and Lactobacillus strains, among others, which can fight against chronic diseases since colonic fermentation produces short chain fatty acids (SCFAs). The present work studied the changes produced in the fibre and polyphenolic compounds during in vitro digestion of gel (AV) and a polysaccharide extract (AP) from *Aloe vera*, after which, these fractions were subjected to in vitro colonic fermentation to evaluate the changes in antioxidant capacity and SCFAs production during the fermentation. The results showed that the phenolic compounds increased during digestion, but were reduced in fermentation, as a consequence, the antioxidant activity increased significantly in AV and AP after the digestion. On the other hand, during in vitro colon fermentation, the unfermented fibre of AV and AP responded as lactulose and the total volume of gas produced, which indicates the possible use of *Aloe vera* and polysaccharide extract as prebiotics.

## 1. Introduction

**Figure 1:**
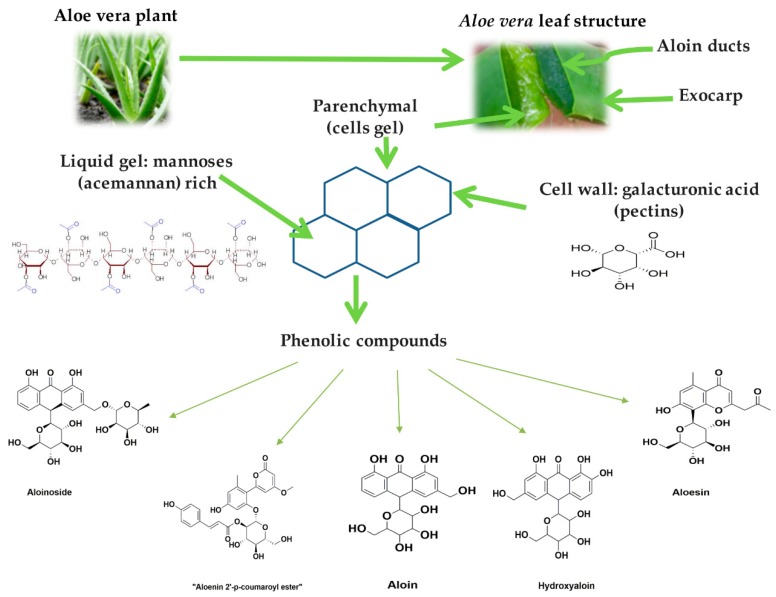
*Aloe vera* plant and its main components.

Plants are a source of a wide spectrum of compounds such as polyphenols, carotenoids, glucosinolates, and lignans, among others. These phytochemicals provide potential beneficial properties to each plant matrix [1]. *Aloe vera* has been long used thanks to its curative and therapeutic properties. It has been reported that only the pulp has more than 75 bioactive compounds [2]. *Aloe vera*, originally from Africa, belongs to the genus *Aloe*, and it is a perennial, succulent xerophyte grown in temperate and sub-tropical regions of the world. *Aloe vera* or *Aloe barbadensis* is part of the *Asphodelaceae* family, of which there are over 360 known species. There are several species under the genus *Aloe*, including *Aloe vera, Aloe barbadensis, Aloe ferox, Aloe chinensis, Aloe indica, Aloe peyrii* etc. Amongst these, *Aloe vera Linn syn. Alo barbadensis Miller* is accepted unanimously as the ideal botanical source of *Aloe* [3]. The pulp or gel of *Aloe vera* is the part of the plant that is of great commercial, pharmacological, alimentary, industrial, and cosmetic importance [3,4]. *Aloe vera* gel contains more than 98% water. The carbohydrate acemannan accounts for greater than 60% of the solid matter found in the gel [5]. Polysaccharides such as acetylglucomannans (acemannans) are glucose-bound mannose units linked by β–(1→4) bonds constituting the backbone of the polysaccharide [6]. Figure 1 shows a representation of *Aloe vera* leaves, which have three portions: (1) yellow sap, constituted mainly of anthraquinones (1,8-dihydroxyanthraquinone derivatives), (2) pulp or parenchymal tissue (mannans, galactans, arabinans, arabinogalactans, pectic substances, and polysaccharides containing glucuronic acid), and (3) exocarp (cellulose, hemicelluloses, and lignin) [4]. Additionally, the gel contains phenolic compounds that can be soluble free or conjugated soluble and insoluble. Conjugated soluble phenolic compounds bind soluble molecules as carbohydrates, proteins, and lipids by esterification in the carboxylic moiety or etherification in the hydroxyl group. Insoluble phenolic compounds generally covalently bind polymers, such as polysaccharides and lignins through an ester bond and are only released from the matrix through acidic, alkaline or enzymatic hydrolysis [7]. It has been reported that these phenolic compounds have properties for the treatment of diabetes, tumours or ulcers, among others [8,9]. Patel et al., using HPTLC, reported the presence of alkaloids, tannins, steroids, sugars, and triterpenes in an extract of *Aloe vera* [10]. 

Soluble or fermentable fibre components, such as mannans, galactans, arabinans, arabinogalactans, and pectic substances, could have prebiotic effects. Prebiotics are carbohydrate-like compounds that can be used in the food industry to modify the composition of microbiota species to benefit human health [11]. Prebiotics mostly target bifidobacteria and lactobacilli, which are two kinds of probiotics [12]. Recent research has suggested that combining both prebiotics and probiotics, namely, symbiotic, can fight chronic diseases since colonic fermentation produces short chain fatty acids (SCFAs). SCFAs are generated as a final product of intestinal microbiota metabolism, mostly out of non-digestible dietary fibre. Acetate (C_2_), propionate (C_3_), and butyrate (C_4_) have different carbon chain lengths. There is research on the beneficial effects of several components of *Aloe vera* on health; however, there are few studies on the changes in fibre and there are no reports available on the estimation of free phenolic during the digestive and fermentative process, where components such as phenolic compounds and the production of SCFAs could have beneficial effects on human health. Therefore, the present work studied the changes in fibre and phenolic compounds produced during in vitro digestion of gel and a polysaccharide extract from *Aloe vera* (AV and AP, respectively), after which these fractions were subjected to in vitro colonic fermentation to evaluate the changes in antioxidant capacity and production of SCFAs during fermentation. 

## 2. Results and Discussion 

### 2.1. Physicochemical Characterization of Aloe Vera Gel (AV)

The pH of AV was 5.4, similar to the value reported by Calderón-Oliver et al., [13]. The total acidity was 0.007 ± 0.02 g/100 g moist sample. The content of total soluble solids (TSS) of AV gel was 0.42 ºBrix. The components present in the TSS can be minerals, vitamins, enzymes, polysaccharides, phenolic compounds, and organic acids, among others [13]. Table 1 shows that the moisture content of the fresh gel extracted from the leaf was the highest value, which agrees with the water activity of the gel and results reported by Hamman [6]. The proteins in the gel could be lectins and lectin-like substances [13].

The dietary fibre content was over 55%, mostly soluble fibre (39.53%), which can be fermented in the colon because it contains soluble oligosaccharides and non-digestible oligosaccharides that could be beneficial to individuals who consume soluble fibre [6]. The percentage of non-fermentable or insoluble AV gel fibre (cellulose, lignin, and hemicellulose) was 15.58 ± 2.04%. When comparing the results against the amount of total dietary fibre (TDF), we observed that AV gel possesses a considerable amount of soluble fibre, which has been reported to provide benefits to diabetes, obesity, gastritis, and neurodegenerative diseases patients who consume it regularly [14]. Interestingly, the polysaccharide content of AV was approximately 71% on a dry basis, which is relevant because polysaccharides have been considered the main component responsible for most of the beneficial properties attributed to the *Aloe vera* plant [15]. The chemical composition may vary with respect to what is reported by other authors, stressing that the ground where the plant grows, the weather, and age of the plant play key roles in the chemical composition of *Aloe vera* [15].

### 2.2. Identification of Phenolic Compounds in AV by UPLC-MS

Six compounds were identified by UPLC-MS in AV (Table 2). Two signals were presented between 4 and 5 min, both with masses of *m/z* 417.18 [M–H]-; these signals were identified as the diastereomers aloin A and aloin B [16,17]. In addition, a peak of *m/z* 555.25 [M – H]- was observed at 4.5 min that could be related to aloenin-2′-*p*-coumaroyl ester. At a retention time of 3.15 min, there was a peak of *m/z* 561.36 [M–H]- that could be the aloinoside isomers [17]. At a retention time of 3 min, a mass of *m/z* 433.19 [M–H]- was observed, which suggests the compound 10-hydroxyaloin, and at 1.54 min, a peak of *m/z* 393.18 [M–H]- was observed and corresponded to aloesin, according to the mass. Table 2 shows the polyphenols identified, aloin A and B, aloesin, 10-hydroxyaloin A or B, and aloenin-2′-*p*- coumaroyl ester, which are anthraquinones reported as powerful stabilizers in oxidative stress [17]. Aloesin is a natural polyphenol originated from *Aloe* plants. Aloesin and/or *Aloe* polysaccharides can reduce systemic oxidative stress by acting directly as potent anti-oxidants and also indirectly by regulating the production of adiponectin and gene expression pathways related to insulin sensitivity, glucose transportation, and fatty acid biosynthesis [18]. 

Aloin A and B are some of the main components of *Aloe vera* gel, which are not only laxatives but are also known to have antioxidant properties at low concentrations [17]. Aloinoside compounds are also anthraquinones, along with aloesin, which exhibits great antioxidant activity and is used to lower oxidative stress indices in diseases such as diabetes [17].

### 2.3. Total Fibre Content of Non-Digestible Fibre Fractions AV-TNDF and AP-TNDF and of Their Soluble (AV-SNDF and AP-SNDF) and Insoluble (AV-INDF and AP-INDF) Fractions

After the digestion process insoluble non-digestible fraction of AV (AV-INDF), insoluble non-digestible fraction of AP (AP-INDF), soluble fraction of the non-digestible fibre of AV (AV-SNDF), soluble fraction of non-digestible fibre of AP (AP-SNDF), were obtained.

The fibre contents of the total non-digestible fibre fraction (TNDF) and its soluble (SNDF) and insoluble (INDF) fractions are shown in Table 3. The INDF content was approximately 25% higher in AP than in AV. The content obtained for AP was comparable to these of nopal cactus (57.7 g/100 g) [19]. The SNDF proportion was 52% higher in AP than in AV, indicating that AP may have a higher amount of partially acetylated mannans as the primary polysaccharide of the gel, although it also contains pectins, some hemicelluloses, mucilages, and gums [4]. Acetylated mannan molecules are mainly responsible for the thick, mucilage-like properties of raw *Aloe vera* gel [4,6]. These results are interesting because *Aloe vera* polysaccharides have been considered the main component responsible for most of the beneficial properties attributed to the *Aloe vera* plant [15]. They are dietary carbohydrates resistant to digestion in the upper GI tract. Due to the human physiological effect of these polysaccharides, they could be labelled as prebiotics, selectively fermented ingredients that allow for specific changes in the composition and/or activity in the gastrointestinal microflora that confer benefits to the hosts wellbeing and health [20]. 

The high fermentability of SNDF produces SCFAs (acetic, propionic, and butyric acids) that contribute to the proper functioning of the large intestine and the prevention of pathologies through their actions in the lumen, colonic and vascular musculature as well as in the colonocyte metabolism [21]. Furthermore, SCFAs reduce intestinal pH and increase water and salt absorption in the large intestine [22]. On the other hand, the fibre contents in AV-INDF and AP-INDF were 26.57 g/100 g and 57.7 g/100 g, respectively. The high proportion of INDF, could increase the stool water content provides bulky/soft/easy-to-pass stools [23].

### 2.4. In Vitro Fermentation 

#### 2.4.1. Changes in pH

**Figure 2:**
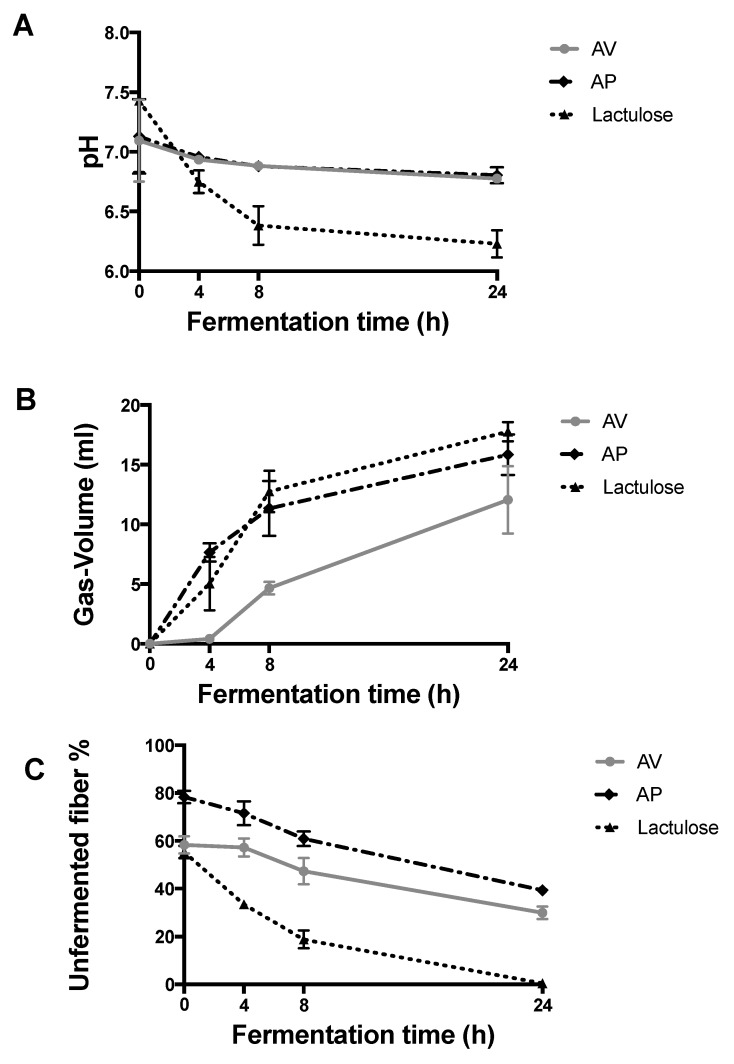
Fermentation kinetics of *Aloe vera* gel (AV) and its polysaccharide extract (AP) during 24 h. (**A**) pH changes during fermentation, (**B**) Volume of gas produced during fermentation, and (**C**) Unfermented residue of samples AV and AP.

Fermentation of TNDF in the large intestine is associated with reduced levels of pH and the consequent proliferation of beneficial microbes such as bifidobacteria [22]. Therefore, changes in pH are often used as indicators of the fermentability of non-digestible matter. Figure 2A shows that the pH decreased with reaction time in both samples: from 7.09 (h0) to 6.76 (h24) in AV and from 7.13 (h0) to 6.79 (h24) in AP. However, the change was gradual when compared to that of the lactulose used as a control. Lactulose has high fermentability and promotes a greater reduction in pH. A significant difference (*p* < 0.05) was observed in the pH of AV and AP with respect to the pH of the lactulose control during the reaction time and up to 24 h of incubation. This behaviour occurs because the control is a disaccharide of fast fermentation [23]. In contrast, AV and AP are matrices mainly composed of non-digestible oligosaccharides or polysaccharides of slow fermentation that produce lower pH [24] or buffer or antacid effects, as Al-Madboly et al. have reported [25].

#### 2.4.2. Volume of gas produced

Gases are produced by microbiota as by-products of their metabolic activities. These microorganisms of the gastrointestinal tract satisfy their energy requirements largely through non-digested carbohydrate fermentation and the subsequent production of SCFAs and certain gas species that include carbon dioxide (CO_2_), methane (CH_4_), and hydrogen (H_2_). The latter is the main by-product of hydrogen fermentation and is generated by hydrogen producers of the phyla Firmicutes and Proteobacteria [26]. Hydrogen gas can be generated from pyruvate cleavage or reoxidation of the reduced pyridine and flavin nucleotides.

Figure 2B shows the volume of gas produced by the microbiota feeding on different components during in vitro fermentation. In AV, the increase in the volume of gas was slow, reaching over 12 mL at 24 h. The volumes indicate that, in the first four hours, fermentation is very slow since, in addition to fermentable fibres of AV, other compounds hamper the beginning of the process, such as insoluble fibres and polyphenols. On the other hand, the AP fraction immediately begins with active fermentation and progressively increases the volume of gas until reaching 15.8 mL (24 h). In this case, the available compounds are mostly soluble polysaccharides, which are rapidly fermentable when compared against the control. The AP extract clearly produced more gases from the beginning, and at 24 h, there was no significant difference with that produced by lactulose (17.7 mL). These results were in agreement with the fibre proportion since the total non-digestible fibre proportion (TNDF) in AP was approximately 50% more than in AV. These results indicate that glucooligosaccharides from AP could be the best carbon source for different microorganisms since they show specific preferences for defined substrates, as reported by Gullon et al. [21]. Gas production was constant throughout the fermentation period, showing a significant difference (*p* < 0.05) against the control at 24 h of fermentation. 

#### 2.4.3. Unfermented fibre (UF)

The TNDF that passes to the large intestine is composed of soluble fibre that is mostly fermented and insoluble fibre, which is mostly not fermented by microorganisms. The latter are residual compounds or UF in the expelled faeces. Figure 2C shows that the UF residues in AV and AP were scarcely fermented, as evidenced by 29.8% and 39.2% of the TNDF for AV and AP, respectively, results which match what was reported by Mudgil and Barak [27].

#### 2.4.4. Quantification of SCFAs 

Table 4 shows the changes in the proportion of SCFAs in AV and AP during fermentation until 24 h, using faeces from healthy volunteers. The SCFAs were quantified as acetate, propionate, and butyrate and were compared with the SCFAs produced by lactulose fermentation, which works as a control given its high fermentability and its consideration as a fermentation standard [21]. Acetate was the main SCFA quantified in both samples, and no significant difference (*p* < 0.05) was found with that produced by lactulose fermentation. This compound is the most common compound in the large intestine [26] and enters the peripheral circulation through peripheral tissues to be metabolized. It has been reported that acetic acid is the main product of bifidobacterial fermentation in a human faecal environment [28]. Acetate is produced by most anaerobes, including acetogens that are able to perform reductive acetogenesis from formate or hydrogen combined with CO_2_ [29]. Acetate plays an important role in controlling inflammation and in combating pathogen invasion [30,31]. 

Propionate is another key SCFA in large intestine fermentation. At 8 h of fermentation, it was produced in similar amounts in the three substrates. However, at 24 h, AV and the control were the substrates that produced the most amount of propionate (3.68 and 4.44 μmol/mg, respectively), which was significantly higher (*p* < 0.05) than that produced by AP (3.12 μmol/mg) at 24 h of fermentation. Propionate, a gluconeogenerator, has been shown to inhibit cholesterol synthesis. On the other hand, after absorption, acetate has been shown to increase cholesterol synthesis [26]; therefore, the higher propionate proportion obtained from AV fermentation could decrease the acetate: propionate ratio and reduce serum lipids and possibly cardiovascular disease risk. The proportion of acetate:propionate in the three samples at 24 h was AV = 2.90, AP = 3.50, and lactulose = 2.57. This indicates that AV and lactulose fermentation produce the highest concentration of propionate. In AV, the proportion was lower than that reported by Gullón et al. [32]. This difference could be due to seasonal changes and geographic locations that lead to significant variations in gel polysaccharides [13]. Producers of propionate largely belong to the phylum Bacteroidetes but also include some Firmicutes.

Finally, AV (1.92 μmol/mg) and AP (1.79 μmol/mg) butyrate yields at 24 h were lower than that of lactulose (4.25 μmol/mg). Butyrate formation occurs in certain Firmicutes bacteria, either via butyrate kinase (in many *Clostridium* and *Coprococcus* species) or via butyric acetate CoA transferase [23]. Butyrate has been studied for its role in nourishing the colonic mucosa and in the prevention of cancer of the colon by promoting cell differentiation, cell-cycle arrest and apoptosis of transformed colonocytes, inhibiting the enzyme histone deacetylase and decreasing the transformation of primary bile acids to secondary bile acids as a result of colonic acidification [26]. Butyric acid exerts a positive influence on the colonic mucosa and affects cell differentiation. It has anti-inflammatory properties and reduces the incidence of colon cancer [29]. Butyrate irrigation (enema) has also been suggested in the treatment of colitis. 

Therefore, SCFA production and their potential delivery to the distal colon due to AV and AP fermentation may result in a protective effect. First, the human large intestine is colonized by dense microbial communities that utilize both diet-derived and host-derived energy sources for growth, predominantly through fermentative metabolism. This highly diverse community has the capacity to perform an extraordinary range of biochemical transformations that go well beyond those encoded by the host genome, and these activities exert an important influence upon many aspects of human health [25]. 

### 2.5. Quantification of Phenolic Compounds in AV and AP and Identification of AV, AV-TNDF, AP, AP-TNDF by HPTLC 

#### 2.5.1. Quantification of Phenolic Compounds in AV and AP

**Figure 3:**
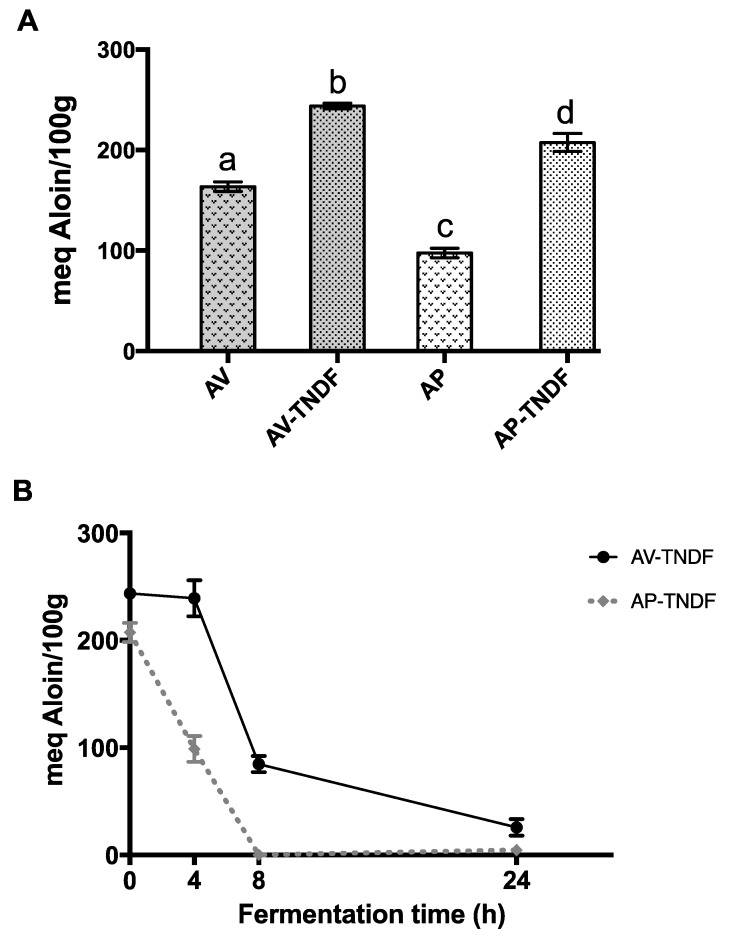
**Total phenolic compounds.** (**A**) Total phenolic compounds before and after digestion of *Aloe vera* gel (AV) and its polysaccharide extract (AP). Where AV and AP correspond to samples before digestion; AV-TNDF and AP-TNDF correspond to samples after digestion. (**B**) Changes in the total content of phenolic compounds during AV-TNDF and AP-TNDF fermentation.

Figure 3A shows the amount of phenolic compounds from AV and AP before and after undergoing an in vitro enzymatic process of digestion simulating the passing of these samples throughout the digestive tract. AV had a higher proportion of phenolic compounds than the extract (AP). In the same figure, a significant increase (*p* < 0.05) of 66% in the amount of phenolic compounds of AV-TNDF with respect to AV was observed, and a significant increase in the amount of phenolic compounds in AP-TNDF compared to AP was also observed, with an increase greater than 100%. However, it is important to note that even when the number of phenolic compounds of AP was lower compared to AV, the sample AP-TNDF had a proportion of phenolic compounds similar to that of AV-TNDF. This may be due to a major release of insoluble phenolic compounds with the acid, alkaline, and enzymatic digestion process, as Anokwuru, et al., have reported [7]. This increase in phenolic compounds, also, could be the result of the opening and breakdown of polysaccharide chains and lattices, liberating most of the phenols when the AV and AP samples undergo the fermentation process [29].

Figure 3B shows the change in the total phenolic compounds before and during fermentation. Fermented products of AV had a stable amount of phenolic compounds during the first 4 h (243.65 to 239.18 meq aloin/100 g), which was reduced at 24 h until 25.72 meq aloin/100 g. For AP-TNDF the fall was higher, starting with a concentration of 207.48 meq aloin/100 g at 0 h fermentation time to 4.58 meq aloin/100 g at 24 h. In humans, insoluble phenolic compounds are released from the matrix in the colon during the fermentation of the ingested material. The release of these phenolic compounds has been identified as beneficial against colon cancer [33]. Free and conjugated phenolic compounds are known as extractable phenolic compounds while bound or insoluble phenolic compounds are known as non-extractable [34]. It is observed that they disappear with fermentation and part of them are degraded, although they remain a constant and significant amount due to the release of insoluble phenolic compounds. There are studies of phenolic compounds that show how the microbiota populations and the phenolic compounds metabolized by this microbiota evolve and change. Cueva et al. (2012) [35] observed a great degradation of phenolic compounds in grape seed extract during in vitro fermentation. Similar results to those obtained in this work. 

#### 2.5.2. Identification of phenolic compounds by HPTLC

Taking into account the results obtained for total phenolic compounds before and after digestion of AV and AP, it was assessed the identification of phenolic compounds in AV, AV-TNDF, AP, and AP-TNDF by HPTLC (Table 5). The standards used were vanillic acid, *p*-hydroxybenzaldehyde, *p*-hydroxybenzoic acid, *p*-cresol and ferulic acid.

In the samples AV, AP, AV-TNDF and AP-TNDF, the presence of ferulic acid and an unidentified compound with Rf’s of 0.05 and 0.04, respectively, was detected. However, with the fermentation process for AV, in the samples FAV4, FAV8 and FAV24, ferulic acid disappeared, and other components appeared with Rf´s of 0.64, 0.57, 0.12 and 0.04. For FPA4, FPA8, and FPA24, only two unidentified compounds with Rf´s of 0.12 and 0.04 appear in small quantities.

Unidentified components can appear when fibre and phenolic compounds are fermented in the intestine. Lee, et al., [36] reported that some bacterial phenolic metabolites were modified in colonic fermentation since specific intestinal bacteria metabolize phenolic compounds to different extents and produce different aromatic metabolites. Changes in intestinal phenolic levels may influence microflora composition. Phenolic compounds are likely to benefit the host by inhibiting pathogen growth and regulating commensal bacteria, including probiotics, and could therefore be considered as prebiotics.

### 2.6. Antioxidant Activity of AV-TNDF and AP-TNDF 

#### 2.6.1. Reducing Power

**Figure 4:**
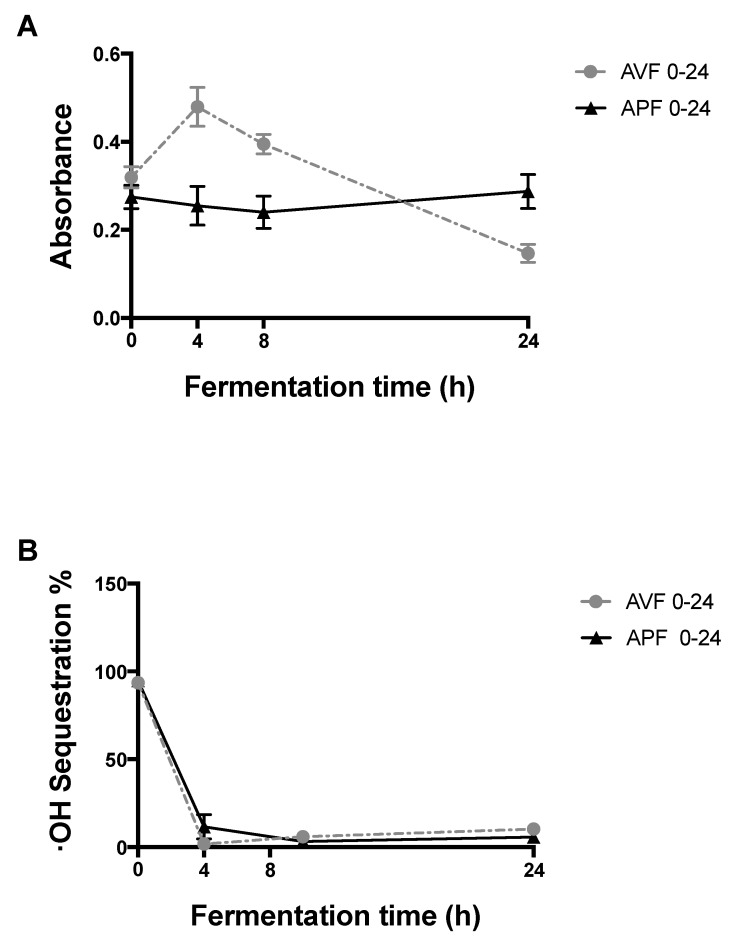
Changes in antioxidant activity during colonic fermentation of *Aloe vera* gel (AV) and its polysaccharide extract (AP). (**A**) Changes in reducing power (Abs 700 nm), due to colonic fermentation. (**B**) Changes in % of sequestration of hydroxyl radical ·OH due to colonic fermentation. (**B**) The results are expressed as the ME ± DS, n = 3.

To establish the appropriate concentration to determine the antioxidant activity of the AV-TNDF and AP-TNDF samples, several concentrations from 0.78 mg/mL to 12.5 mg/mL were evaluated. It was found that the samples had a concentration-dependent behaviour, with 12.5 mg/mL being the one with the highest activity (*p* < 0.05) (data not shown); therefore, the tests of reducing power and radical ·OH sequestration correspond only to this concentration. Figure 4A shows the changes in the reducing power during 24 h of the fermentation kinetics at 12.5 mg/mL of the fraction of non-digestible *Aloe vera* (AV-TNDF) and the non-digestible fraction of *Aloe vera* polysaccharide extract (AP-TNDF). The highest activity of reducing power of AV-TNDF was observed at 4 h (AVF4). This indicated that, in general, fractions derived from AV show a greater reducing power than fractions from AP. A possible explanation is that AV-TNDF had higher concentrations of inactive polyphenols in their matrices that can interact with the medium after gastrointestinal digestion. Acids and enzymes can open the fibres containing the polyphenols, which in turn are more bioavailable at the end of digestion. These results agree with the identification of the phenolic compounds shown in Table 5. The appearance of the unidentified compounds with Rf´s of 0.64, 0.57 and 0.12 may have greater reducing power than those identified in AP-TNDF. 

The fermentation samples of AP-TNDF (AP0-24) had antioxidant capacities and reducing powers that experience limited change over time. As shown in Table 5, during fermentation, AP-TNDF lost unidentified phenolic compounds, which leads to a decrease in reducing power, and a correlation between the antioxidant capacity and the reducing power was seen, as Alugoju et al. have reported for certain bioactive compounds [37]. The reducing power of 0.2 of inhibition was reported with methanolic extracts from non-digested fresh samples [28]. Cueva et al. [35] demonstrated the appearance of new phenolic compounds that were not present or were present in small amounts before fermentation such as hydroxyphenylacetic acid, phenylpropionic acid or phenylacetic acid with colonic in vitro fermentation of grape seed extract.

#### 2.6.2. OH Radical Sequestration

The statistical analysis of ·OH radical sequestration revealed that all extracts, except AP, were dose dependent. The elimination of hydroxyl radicals from fractionated polysaccharides and AV gel extracts has been reported by Chun-Hui et al. [38]. Figure 4B shows the efficacy of 12.5 mg/mL solutions of AV (35.47%), AV-TNDF (93.54%), AP (3.07%) and AP-TNDF (94.78%) to sequester hydroxyl radicals. The results of AV are even lower than assays reported by authors such as Ray et al. [39], who report values of up to 48.01% using methanolic extracts of fresh *Aloe vera* gel without a digestion process. This difference may be due to the age of the plant and edaphoclimatic conditions as Sánchez-Machado et al., have reported [40]. The higher efficacy of AV-TNDF and AP-TNDF could be due to the increase in phenolic compounds, because fresh samples have large concentrations of inactive polyphenols in their matrices that can interact with the medium after a digestion process such as gastro intestinal digestion. Additionally, it could be the result of the opening and breakdown of polysaccharide chains and lattices, liberating most of the phenols when the AV and AP samples undergo the fermentation process [29].

Figure 4B shows the ∙OH sequestration percentage of the synthesized or released compounds in solutions of fermented AV (AVF) and the fermented polysaccharide extract (APF) at 12.5 mg/mL. At this concentration, the ferments exhibited a decrease in antioxidant activity during the first 4 h of fermentation in the two samples up to 1.85% (AVF4) and 11.52% (APF4). The AVF antioxidant activity experienced a slight increase until 24 h of fermentation with 7.76% effectiveness observed for AVF24, and the APF antioxidant activity remained low and constant, reaching 5.74% effectiveness at 24 h. 

In contrast to the reducing power, in this case, the percent of ·OH sequestration drops dramatically over time within the first few hours of fermentation. In this case, the phenolic or other compounds that have effective ·OH sequestration in the undigested fractions AV-TNDF and AP-TNDF are rapidly degraded by the enzymes, bacteria or fermentation conditions, drastically lowering the antioxidant activity. Lee et al., have reported a decrease in concentration after incubation with a faecal bacteria homogenate in compounds present in the tea extract, including epicatechin, catechin, 3-O-methyl gallic acid, gallic acid, and caffeic acid [36]. The difference in the behavior of reducing power and ·OH radicals are because they have different ways of sequestering Reactive Oxygen Species (ROS). ·OH radicals react with biomolecules such as lipid, protein and DNA [41].

## 3. Materials and Methods 

### 3.1. Raw Material

Leaves of *Aloe vera* plants (aged 3–4 years) were obtained from a market in Mexico City. The leaves were washed with soap and water, followed by disinfection with ethanol. The gel was extracted by removing the cortex and was frozen and lyophilized until use.

### 3.2. Physicochemical Characterization of Aloe Vera (AV)

In the raw gel of *Aloe vera*, the acidity was determined using AOAC method 939.05 (2015) [42], while the pH was determined using a potentiometer (pH-120; Conductronic, Puebla, Pue, Mexico). Soluble solids (ºBrix) were assessed using a digital refractometer (HSR-500; Atago, Kobe, Hyogo-ken, Japan) at a 0–32 ºBrix scale considering that 1 ºBrix = 1 g soluble solid in 100 g of solution [43]. Moisture was determined according to method 2005.02, raw protein by the Kjeldahl method (2005.06), raw fat by a Soxhlet method (920.39c), and ash by method 923.03 (AOAC, 2015) [42]. Neutral detergent fibre was assessed in the lyophilized sample of the gel, according to the 2002:04/ISO technique (16472:2005) from AOAC [42]. Total dietary fibre was determined through the enzymatic-gravimetric method (32–05) outlined by AOAC (2015) [42].

### 3.3. Identification of phenolic Compounds by Ultra-Performance Liquid Chromatography-Tandem Mass Spectrometry (UPLC-MS) in Aloe Vera (AV)

The identification of phenolic compounds in the gel of *Aloe vera* (AV) was performed with ultra-performance liquid chromatography (UPLC) by utilizing an Acquity UPLC H-Class (Waters Corp, Milford, MA, USA) coupled with a Waters Xevo G2-X2 Tof system with an electrospray ionization (ESI) interface. The conditions for the chromatographic analysis were as follows: the mobile phase consisted of 0.1% formic acid in water/acetonitrile at a flow rate of 0.3 mL/min and 35 °C. A BEH C18 Column (2.1 mm X 100 mm) was used and the chromatograms were registered through detection of the Total Ion Current. The MS conditions of analysis in negative-ion mode were as follows: drying gas (nitrogen) flow rate, 8 L/min; gas temperature, 180 °C; scan range, *m/z* 50–3000; capillary voltage, 4500 V; and nebulizer pressure, 2.5 bar [44]. A solution of 8 mg/mL of lyophilized AV was prepared, homogenized, and passed through a 0.22 µm cellulose filter prior to analysis.

### 3.4. Extraction of Polysaccharides (AP) from Aloe Vera Gel

The extraction of *Aloe vera* gel polysaccharides (AP) followed a modification of the technique reported by Ni et al. [4]. One gram of lyophilized *Aloe vera* gel was suspended in 80 mL of a 95% water/methanol solution, homogenized with a Polytron, and centrifuged at 936× *g* (Allegra 64R; Beckman, Pasadena, CA, USA) for 10 min. The first precipitate was stored, and the supernatant was centrifuged one more time at 10,285× *g* for 15 min. The first and second precipitates were combined, re-suspended in deionized water and lyophilized. The sum of these two fractions was the polysaccharide extract (AP). The first precipitate corresponds to the cell wall fibre, and the second precipitate corresponds to micro-particles from cell organelles of *Aloe vera* [4]. 

### 3.5. Indigestible Fraction and in Vitro Fermentation

#### 3.5.1. Indigestible Fermentation of Aloe Vera Gel (AV) and Polysaccharides (AP)

**Figure 5:**
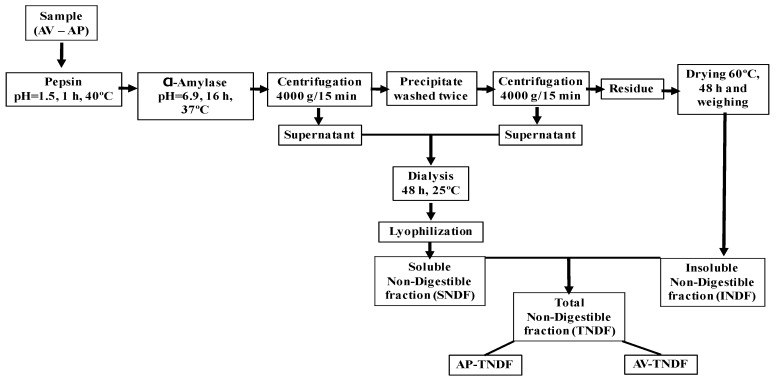
Indigestible fraction. The obtaining of indigestible fractions of AV and AP. AV= *Aloe vera gel, AP= Aloe* polysaccharides; AV-NDF = Insoluble non-digestible fraction of AV, AP-NDF = Insoluble non digestible fraction of AP, AV-SNDF = Soluble fraction of the non-digestible fibre of AV, AP-SNDF = Soluble fraction of non-digestible fibre of AP.

Figure 5 shows the flowchart of the isolation of the indigestible fibre fractions of AV and AP The indigestible fraction (IF) of (AV) and (AP) (Figure 5) was determined following the method proposed by Saura-Calixto et al. [45] with some modifications. Briefly, 300 mg of both fractions were suspended in HCl-KCl buffer (pH 1.5). Then, 0.2 mL of a pepsin solution at a concentration of 300 mg/mL in HCl-KCl buffer (>250 units/mg, P7000; Sigma Aldrich, Toluca, Edo. Mex. Mexico) was added and incubated at 40 °C for 1 h with constant stirring to simulate the digestive process. 

To simulate small intestine digestion, pH was adjusted to 6.9, and 9 mL of tris-maleate buffer (0.1 M) was added, followed by 1 mL of α-amylase solution (3480 units/mL, A3176; Sigma Aldrich), at a concentration of 120 mg/mL in tris-maleate buffer. The mix was incubated at 37 °C for 16 h at constant stirring. Lipid digestion enzymes such as lipase or biliary juices were omitted given that the amount of fat (lower than 4%) was not significant. After the reaction time, the samples were centrifuged at 4000× *g* for 15 min, and the supernatants were saved. The precipitate was washed twice with 10 mL of distilled water and centrifuged; supernatants were collected in 80 mL beakers. The tubes containing the solid residue were placed in an oven at 60 °C for 48 h, and the residue was then weighed as the amount of insoluble non-digestible fibre in AV (AV-INDF) and AP (AP-INDF). Both insoluble non-digestible fibre fractions were stored at 4°C until the fermentation experiments. 

The supernatant was placed on dialysis membranes previously treated with boiling water for 20 min. The supernatant was dialyzed (cellulose membrane 12000–14000 Dalton MWCO-18) at a constant water flow of 7 L/h (30 mL/15 sec) at 25 °C for 48 h. Finally, the content of the membranes was lyophilized and weighed to obtain the soluble fraction of the non-digestible fibre of AV (AV-SNDF) and non-digestible fibre of AP (AP-SNDF); these fractions were stored at 5 °C until use in the fermentation process [46]. 

#### 3.5.2. In Vitro Fermentation of AV-TNDF and AP-TNDF 

The samples obtained in the digestion, which were the soluble fraction (SNDF) and the insoluble fraction (INDF), were combined as the total non-digestible fraction (TNDF) of AV and AP to be fermented. In vitro fermentation was assessed in the AV-TNDF and AP-TNDF fractions. Fermentation was prepared according to a modified version of Martín-Carrón and Goñi [46]. The samples were placed in a culture system under strict anaerobic conditions for 24 h. Human faeces were used as inocula, and anaerobic conditions were maintained using oxygen-free carbon dioxide.

##### Inoculum

To obtain the 10% *w/v* inoculum, the faeces of four volunteers were weighed and placed in beakers containing a sterile and anaerobic fermentation medium. The inocula were mixed in a Stomacher 80 Lab blender (Seward Medical, London, UK for 10 min and then filtered (1-mm mesh) before use. 

##### Medium

A micromineral solution was prepared using CaCl_2_-2H_2_O, MnCl_2_-4H_2_O, CoCl_2_-6H_2_O, and FeCl_3_-6H_2_O mixed in a buffer solution ((NH_4_)HCO_3_ and NaHCO_3_) in distilled water. A macromineral solution containing Na_2_HPO_4_, KH_2_PO_4_, and MgSO_4_-7H_2_O was made in distilled water, as was a reducing solution containing cysteine hydrochloride (C-1276, Sigma-Aldrich, Toluca, Edo. Mex., Mexico).

##### Procedure

Substrate (100 mg) of the total non-digestible fraction (AV-TNDF or AP-TNDF) was weighed in a 60 mL serum vial (Supelco; Bellefonte, PA, USA), and 8 mL of fresh medium and 2 mL of inoculum were added. Vials were sealed with rubber stoppers and placed in a water bath with constant stirring at 37 °C. The fermentation kinetics was monitored at 0, 4, 8 and 24 h to obtain fermented samples at each time point (AVF4, AVF8 and AVF24 or APF4, APF8 and APF24). A control for each time containing lactulose (L-7877, Sigma-Aldrich, Toluca, Edo. Mex., Mexico) and a target without substrate were included as controls.

Gas volume production and pH were measured at 0, 4, 8 and 24 h. Fermentation was stopped by adding 2.5 mL of1 M NaOH. Samples were centrifuged at 2500× *g* for 10 min, and 3 mL of supernatant was taken in duplicate for SCFA determination by gas chromatography.

##### Unfermented Fibre

The precipitate of each tube from the fermentation kinetics experiment was homogenized in 50 mL of 0.9% NaCl for 3 min. All precipitates were filtered using Dacron filters (pore size 50 μm), which were dried to a constant weight before use. Unfiltered residues were washed twice with 50 mL of 0.9% NaCl and washed twice with 5 mL of acetone to eliminate mineral and inorganic residues in addition to lipids and other hydrophobic compounds. Filter papers were dried at 60 °C to constant weight, assessing the unfermented fibre (UF) [47] that was calculated by the following formula:

UF = weight (filter + washed residue) − initial filter weight.(1)

Then, UF(AV-TNDF) and UF(AP-TNDF) were obtained.

#### 3.5.3. Quantification of SCFAs in AV-TNDF and AP-TNDF 

SCFA quantification was carried out in AV-TNDF, AP-TNDF and lactulose fermented according the methodology reported by Saura-Calixto et al., [45] and adapted from Zhao et al., [48]. Briefly, 500 µL of supernatant of each fermentation time sample was mixed with 400 mL of 2-methylvaleric acid as an internal standard (10987-8; Sigma, Toluca, Edo. Mex., Mexico) and 100 mL of HClO_4_ to maintain a constant pH in the samples. The mixture was centrifuged at 10,000 at 4 °C for 15 min, and the supernatant was placed in gas chromatography vials. A Clarus 500/580 GC gas chromatographer (Perkin-Elmer, Inc. Shelton, CO., USA) was used with a TG-WAXMS-A GC column (Thermo Fisher Scientific, CDMX, Mexico) and a flame ionization detector with an injector temperature of 270 °C and a detector temperature of 300 °C. Glacial acetic acid, propionic acid, butyric acid, and 4-methylvaleric acid (Sigma, 320099, 94425, 19215 and 10987-8) were used as standards to obtain calibration curves and assess retention times. The oven was heated to 95 °C at 2 min and increased at 120 °C/min to 240 °C; the carrier flow was 1 mL/min, and the carrier gas flow was 20 mL/min.

#### 3.5.4. Quantification of Phenolic Compounds 

Total phenolic compounds were quantified using the Folin–Ciocalteu method [49] in the AV, AP, AV-TNDF, AP-TNDF, AVF4, AVF8, AVF24, APF4, APF8 and APF24 samples. The samples were homogenized in 0.5 mL methanol, added Folin-Ciocalteu reagent, and saturated sodium carbonate solution and water. After 60 min, the absorbance was measured at 760 nm. A calibration curve was performed with aloin, because it is the most representative phenolic compound in *Aloe vera* [50]. The concentration of total phenolic compounds was expressed as milligram equivalents of aloin/100 g sample. 

#### 3.5.5. Identification of Phenolic Compounds by HPTLC

HPTLC was accomplished according to a modified version of the methodology published by Paillat, et al., [51]. At the beginning volumes of standard solutions of ferulic acid, *p*-hydroxybenzaldehyde (PHB), *p*-hydroxybenzoic acid (APHB), *p*-cresol, *p*-creosol, and vanillic acid (722820, 54590, 54630, 61030, 41340, and 68654, Sigma-Aldrich, Química S.A. de C.V.) were applied at a concentration of 3 mg/mL. After that, were applied 15 μL of AV, AV-TNDF, AP, AP-TNDF and the kinetic fermentation samples (AVF4, AVF8, AVF24 and APF4, APF8, APF24) at a concentration of 12.5 mg/mL. Samples were placed on TLC silica gel 60 F254 plates (E. Merck, Darmstadt, Germany) using an ATS 4 TLC sampler (CAMAG, Muttenz, Switzerland) at a constant application rate of 120 nL s^−1^ and developed in a CAMAG automated developing chamber ADC2 (47% moisture) saturated and preconditioned for 5 min to a 50-mm distance with an *n*-hexane: chloroform:methanol:acetic acid solvent system (5:36:4:0.5 *v/v/v/v*). Plates were scanned at 254 nm and 366 nm in a CAMAG TLC III scanner (slit size 4 mm × 0.3 mm) at a scanning speed of 10 mm s^−1^ and a data step resolution of 50 μm. 

### 3.6. Antioxidant Activity

#### 3.6.1. Reducing Power and Hydroxyl Radical Scavenging Activity

The reducing power of AV, AV-TNDF, AP, and AP-TNDF was assessed by the Oyaizu method [52]. Various concentrations of methanolic extracts (12.5, 6.25, 3.12, 1.56, and 0.78 mg/mL) were mixed with 2.5 mL 200 mmol/L sodium phosphate buffer (pH 6.6) and 2.5 mL 1% potassium ferricyanide. The mixture was incubated at 50 °C for 20 min. After 2.5 mL of 10% trichloroacetic acid (*w/v*) were added, the mixture was centrifuged at 650 rpm for 10 min. The upper layer (5 mL) was mixed with 5 mL deionised water and 1 mL 0.1% of ferric chloride; the absorbance was measured at 700 nm, a higher absorbance indicating a higher reducing power. The assays were carried out in triplicate and the results were expressed as mean values ± standard deviations. Finally, the reducing power in the kinetic fermentation samples (AVF4, AVF8, AVF24, APF4, APF8 and APF24) was determined but only with a concentration of 12.5 mg/mL. This was the concentration that gave the best results in the previous samples and was chosen for this second part of the work. 

#### 3.6.2. ∙OH Radical Sequestration

To determine the scavenging activity of the hydroxyl radical (∙OH), the method described by Li et al. [53] was applied. The antioxidant activity of AV, AV-TNDF, AP and AP-TNDF was assessed at 12.5, 6.25, 3.12, 1.56 and 0.78 mg/mL. Both 1,10-phenanthroline (0.75 mM) and FeSO4 (0.75 mM) were dissolved in phosphate buffer (pH 7.4) and mixed thoroughly. To start the reaction, H_2_O_2_ (0.01%) and samples were added. The mixture was incubated at 37 °C for 60 min and the absorbance was measured at 536 nm. Finally, the scavenging activity of the hydroxyl radical in the kinetic fermentation samples (AVF4, AVF8, AVF24, APF4, APF8 and APF24) was determined but only with a concentration of 12.5 mg/mL his was the concentration that gave the best result in the previous samples and was chosen for this second part of the work. 

### 3.7. Statistical Analysis

Differences between experimental groups were analysed by one-way ANOVA and Tukey’post hoc test for repeated samples. To compare the maximum and minimum values of the total SCFA concentration, the data were analysed by a paired one-tailed Student’s t-test. Data were processed using GraphPad Prism 6.0 (GraphPad Software Inc., San Diego, CA, USA). Values of *p* ≤ 0.05 were considered statistically significant. 

## 4. Conclusions

*Aloe vera* gel (AV) and polysaccharide extract of *Aloe vera* (AP) are a matrix mainly composed of non-digestible oligosaccharides or polysaccharides of slow fermentation, since that produces a lower pH. The behavior of AV and AP during in vitro colon fermentation was similar to that of lactulose, what indicates the possibility of using *Aloe vera* and polysaccharide extracts as prebiotics. The SCFA production and their potentially delivery to the distal colon due to AV and AP digestion and fermentation process, may result in a protective effect, firstly of human large intestine, since this could be colonised by dense microbial communities that utilized both diet-derived and host-derived energy sources for growth predominantly through fermentative metabolism. In the same way, the antioxidant activity also increases significantly in both the reducing power test and the ·OH radical sequestration when going from AV and AP to its indigestible fraction AV-TNDF and AP-TNDF. Finally, we can conclude that there were no significant differences during the digestion and fermentation of *Aloe vera* and its extract despite the fact that the content of dietary fibre in AP was significantly higher than that of *Aloe vera* gel. During in vitro colon fermentation, the unfermented fibre of AV and AP had a similar response to that of lactulose, as well as the total volume of gas produced, which indicates that *Aloe vera* and polysaccharide extract can possibly be used as prebiotics.

## Figures and Tables

**Table 1:** Chemical composition of *Aloe vera* ^1^.

COMPONENT	AMOUNT (g/100g db)
Moisture	97.50 ± 0.04
Proteins	2.15 ± 0.08
Lipids	3.27 ± 0.85
Ashes	12.62 ± 1.1
Total Dietary Fibre (TDF)	55.11 ± 0.55
Neutral Detergent Fibre (NDF)	15.58 ± 2.04
Nitrogen-free extract	26.85 ± 1.1

^1^ Determinations were made in triplicate and the results are expressed in dry basis as the mean ± SD.

**Table 2:** Phenolic compounds identified in the aqueous extract of *Aloe vera* by UPLC-MS.

PEAK	Rt ^a^ (min)	*m/z* [M-H]^−^	*m* [M]	Tentative Identification
**1**	1.52	393.18	394.18	Aloesin
**2**	3.15	433.19	434.19	10-Hydroxyaloin
**3**	3.15	561.36	562.36	Aloinoside
**4**	4.45	417.19	418.19	Aloin A
**5**	4.50	555.25	556.25	Aloenin-2′-*p*-coumaroyl ester
**6**	4.68	417.19	418.19	Aloin B

^a^ Retention time.

**Table 3:** Non-digestible fraction of *Aloe vera* gel (AV) and its polysaccharide extract (AP).

Sample	SNDF (g/100g)	INDF (g/100g)	TNDF (g/100g)
AV	20.57 ^a^ ± 0.83	26.57 ^a^ ± 0.02	47.14
AP	39.34 ^b^ ± 0.55	53.05 ^b^ ± 0.40	92.39

SNDF = Soluble non digestible fraction; INDF = Insoluble non digestible fraction; TNDF = Total non-digestible fraction. The results are the average of three repetitions ± SD. Means between rows with different letters (a and b) are significantly different for a significance level of (*p* ≤ 0.05) by t-student test.

**Table 4:** Concentration of SCFA (μmol/mg) produce during the fermentation of *Aloe vera* gel (AV), its polysaccharide extract (AP) and lactulose as a carbon source

Fermentation Time	Acetic Acid			Propionic Acid			Butyric Acid			Total SCFA		
	AV	AP	Lactulose	AV	AP	Lactulose	AV	AP	Lactulose	AV	AP	Lactulose
0	2.16 ± 1.07 ^b^	0.93 ± 0.36 ^a^	2.16 ± 0.08 ^b^	0.09 ± 0.05 ^a^	0.14 ± 0.03 ^b^	0.09 ± 0.03 ^a^	0.09 ± 0.04 ^b^	0.14 ± 0.01 ^c^	0.05 ± 0.01 ^a^	2.33 ± 1.08 ^ab^	1.21 ± 0.39 ^a^	2.34 ± 0.11 ^b^
4	3.15 ± 0.55 ^a^	3.20 ± 0.64 ^a^	4.50 ± 0.73 ^a^	1.32 ± 0.26 ^a^	1.30 ± 0.14 ^a^	1.07 ± 0.06 ^a^	0.31 ± 0.05 ^a^	0.63 ± 0.04 ^c^	0.47 ± 0.02 ^b^	4.77 ± 0.84 ^a^	5.13 ± 0.80 ^a^	5.15 ± 0.93 ^a^
8	6.12 ± 0.66 ^a^	7.00 ± 0.26 ^a^	9.75 ± 1.92 ^b^	2.50 ± 0.27 ^ab^	2.16 ± 0.32 ^a^	3.42 ± 0.90 ^b^	0.79 ± 0.04 ^a^	1.02 ± 0.13 ^b^	2.09 ± 0.03 ^c^	9.41±0.96 ^a^	10.18 ± 0.70 ^a^	12.51 ± 2.79 ^a^
24	10.67 ± 0.3 ^a^	10.93 ± 1.33 ^a^	11.43 ± 1.57 ^a^	3.68 ± 0.24 ^b^	3.12 ± 0.14 ^a^	4.44 ± 0.69 ^b^	1.92 ± 0.10 ^a^	1.79 ± 0.14 ^a^	4.25 ± 1.12 ^b^	16.27 ± 0.60 ^a^	15.83 ± 1.47 ^a^	17.03 ± 1.51 ^a^

Standard deviation for n = 3. Different letters (a and b) indicate significant differences (*p* < 0.05) between columns for the same acid.

**Table 5:** Identification of phenolic compounds of AV, AV-TNDF, AP, AP-TNDF and their fermented fractions obtained by HPTLC.

Phenolic Compounds	Colour	Rf	AV	AV-TNDF	AP	AP-TNDF	FAV4	FAV8	FAV24	FPA4	FPA8	FPA24
Vanillinic acid	Dark blue	0.54	-	-	-	-	-	-	-	-	-	-
*p*-Hydroxybenzaldehyde	Dark blue	0.65	-	-	-	-	-	-	-	-	-	-
*p*-Hydroxybenzoic acid	Dark blue	0.45	-	-	-	-	-	-	-	-	-	-
*p*-Cresol	Dark blue	0.72	-	-	-	-	-	-	-	-	-	-
*p*-Creosol	Dark blue	0.86	-	-	-	-	-	-	-	-	-	-
Ferulic acid	Light blue	0.55	+	+	+	+	-	-	-	-	-	-
Unidentified	Violet	0.64	-	-	-	-	++	++	++	-	-	-
Unidentified	Yellow	0.57	-	-	-	-	++	++	++	-	-	-
Unidentified	Yellow	0.12	-	-	-	-	++	++	++	+	+	+
Unidentified	Red	0.06	++	+	-	-	-	-	-	-	-	-
Unidentified	Light blue	0.04	+	+	++	+++	+	+	+	+	+	+

AV = *Aloe vera*, AV-TNDF = non-digestible fraction of AV, AP= AV polysaccharides, AP-TNDF= non-digestible fraction of AP and their fermented fractions obtained by HPTLC. Samples obtained at 4, 8 and 24 (FAV4, FAV8, FAV24, FPA4, FPA8, FPA24). The symbols indicate: +++ highly concentrated compound, ++ compound concentrate, + little concentrated compound, - no appreciable concentration of the compound. HPTLC, remission at 366 nm. CAMAG.
